# Building a PET/CT Program in Sub-Saharan Africa: A Health-System Framework for Financing, Affordability, and Sustainable Implementation

**DOI:** 10.3390/cancers18213533

**Published:** 2026-11-03

**Authors:** Paul N. Mobit, Waleed F. Mourad, Wilfried B. Mosse, Lana M. Khair, Christopher F. Njeh, Edmund Folefac

**Affiliations:** 1 Department of Radiation Oncology, University of Mississippi Medical Center, Jackson, MS 39213, USA; pmobit@umc.edu; 2 Cameroon Oncology Center, Douala P.O. Box 1882, Cameroon; mossleboss2@gmail.com (W.B.M.); ajongkob@gmail.com (L.M.K.); 3 Cameroon Cancer Foundation, Douala P.O. Box 1882, Cameroon; 4 Department of Radiation Medicine, University of Kentucky College of Medicine, Markey Cancer Center, Lexington, KY 40536, USA; waleed.mourad@uky.edu; 5 Department of Radiation Medicine, Indiana University, Indianapolis, IN 46202, USA; 6 Department of Medical Oncology, James Comprehensive Cancer Center, Ohio State University, Columbus, OH 43210, USA; edmund.folefac@osumc.edu

**Keywords:** PET/CT, fluorine-18 FDG, cyclotron, molecular imaging, health economics, health financing, cancer systems, Sub-Saharan Africa, Cameroon

## Abstract

**Simple Summary:**

PET/CT can improve cancer staging, treatment planning, response assessment, and follow-up, but establishing a PET/CT service in a resource-constrained health system requires much more than purchasing a scanner. This study develops a transparent planning framework using Cameroon as an illustrative national case. It links cancer burden, pathology capacity, guideline-based referral, staffing, equipment availability, financing, and patient throughput to the cost of providing PET/CT. The model separates fixed, step-fixed, and variable costs; makes the financing equations explicit; and distinguishes economic cost from public subsidy, reimbursement, and patient payment. The analysis shows that PET/CT is most feasible when pathology and referral pathways generate sufficient demand, equipment is reliably maintained, and public or concessional financing limits the amount that must be recovered from patients. The framework is intended to support governments and cancer centers planning sustainable molecular imaging services in Sub-Saharan Africa.

**Abstract:**

**Background**: PET/CT is integral to modern oncology, yet access remains limited in Sub-Saharan Africa (SSA) because of capital requirements, radiopharmaceutical production complexity, workforce constraints, and weak reimbursement systems. We evaluated the modeled financial feasibility of a cyclotron-based PET/CT program using Cameroon as an illustrative national case and reframed implementation as a health-system planning problem rather than an equipment-purchase decision. **Methods**: A deterministic scenario model was developed over a 10-year horizon. The model was aligned conceptually with IAEA PET/cyclotron planning guidance, WHO OneHealth ingredients-based costing, and ISPOR principles for transparent decision modeling. The entry-level configuration comprised one PET/CT scanner, an 11–13 MeV negative-ion medical cyclotron, F-18 targetry, two synthesis modules, hot cells, a quality-control laboratory, backup power, and supporting infrastructure. Capital investment was modeled at USD 9 million as a planning estimate, with wide sensitivity analysis. Operating costs were separated into fixed, step-fixed, and variable components. Annual completed scan volumes of 500–5000 were evaluated, together with financing at 3%, 6%, 10%, and 12% APR, public capital support, operating subsidy, equipment availability, disease-specific demand, and alternative generator/satellite strategies. **Results**: At 6% financing, annual debt service for USD 9 million was USD 1.22 million. Modeled operating expenditure rose with activity from approximately USD 0.93 million at 500 completed scans to USD 1.77 million at 5000 scans because staffing and consumables were activity dependent. Total economic cost per completed scan declined from approximately USD 4311 at 500 scans to USD 598 at 5000 scans. If government provided the capital infrastructure, the operating cost to be recovered declined from USD 1865 to USD 353 per scan over the same activity range. A disease-specific incident-case model based on GLOBOCAN 2022 data for Cameroon showed that pathology confirmation, clinical eligibility, and referral completion materially constrain demand; at 30% histologic confirmation and 80% referral completion, approximately 1005 incident-case PET/CT examinations were generated under the base planning assumptions. Equipment availability of 80%, 90%, and 95% materially changed cost per completed scan in a 3000-slot program. **Conclusions**: Cyclotron-based PET/CT programs may achieve financial sustainability when patient throughput is sufficient, financing is appropriate, operations are reliable, and the surrounding cancer system can generate and act on evidence-based referrals. The model provides a transparent planning framework rather than empirical proof of sustainability and should be locally calibrated before investment decisions.

## 1. Introduction

Sub-Saharan Africa (SSA) is projected to experience a rapid increase in cancer incidence as a result of population growth, ageing, urbanization, and epidemiological transition. Strengthening diagnostic and treatment infrastructure is therefore an important component of national cancer-control planning [1,2]. Positron emission tomography/computed tomography (PET/CT) combines metabolic and anatomical information and supports tumor detection, staging, radiotherapy target delineation, response assessment, and surveillance. Fluorine-18 fluorodeoxyglucose (18F-FDG) remains the most widely used PET radiotracer and has guideline-recognized applications across multiple malignancies [3]. Access to PET/CT is not available to more than 95% of the population in Sub Saharan Africa as only Nigeria, Kenya, South Africa, and Ghana have PET/CT centers utilizing 18F-FDG, which required a cyclotron to produce. Establishing a service requires not only a scanner but also radiopharmaceutical supply, shielding, hot cells, quality control, radiation-safety systems, reliable utilities, specialized staff, and maintenance capability. Also, 18F-FDG has a short physical half life of approximately 110 min, and this makes production scheduling, transport time, and the coverage zone limited to a few hours [4,5,6]. Douala and Yaoundé are metropolis in Cameroon with a population above 3 million each and are separated by approximately 250 km, but road transport may require several hours; this limits the practical distribution radius of short-lived radiopharmaceuticals and increases the attractiveness of either patient-referral hubs or carefully planned satellite distribution networks [7,8,9]. The Republic of Cameroon does not really have a public insurance program so that many families have pay 100% out of pocket for direct health spending, making the distinction between economic cost, reimbursement, public subsidy, and patient price particularly important for sustainability [10,11,12,13,14].

**Figure 1:**
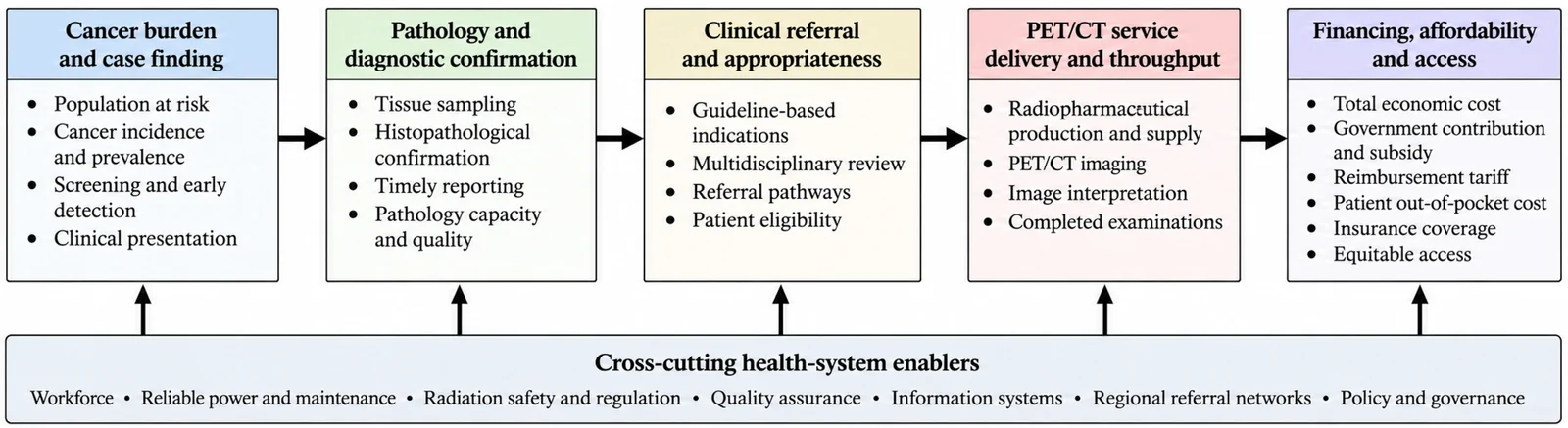
Health-system pathway for sustainable PET/CT implementation.

Most publications on PET/CT in Africa describe service availability, clinical applications, or broad infrastructure gaps. Fewer studies explicitly connect financing structure, activity-dependent operating costs, pathology capacity, referral losses, staffing, equipment availability, and treatment readiness in a reproducible planning model. The purpose of this study was therefore to develop a deterministic framework for a national PET/CT imaging center using Cameroon as an illustrative case. The conceptual premise is that cancer incidence does not translate directly into PET/CT demand. Patients must enter the health system, undergo appropriate diagnostic evaluation and tissue confirmation when indicated, reach a clinician familiar with evidence-based PET/CT indications, obtain referral and financially capable to complete the examination, and have access to treatment that can act on the result. Failure at any upstream stage reduces scanner utilization and increases average cost [15,16,17]. This description is illustrated in Figure 1.

## 2. Materials and Methods

### 2.1. Study Design, Perspective, and Model Alignment

We performed a deterministic scenario-based financial feasibility analysis from provider and health-system perspectives. The study is a strategic planning model, not a patient-level cost-effectiveness analysis as it does not take into consideration other costs incurred by the patient, such as transport, laboratory testing, productivity losses, caregiver costs, etc. The model architecture was cross-checked against two established planning traditions. First, the IAEA Planning Guide entitled Clinical PET Centre and Technical Reports Series No. 471, which specifies the major facility, cyclotron, radio-pharmacy, staffing, quality-control, and radiation-safety components that should be considered when establishing a PET/cyclotron service [5,9]. Second, the WHO OneHealth approach uses ingredients-based costing in which quantities of resources are multiplied by prices and allowed to change with scale and time [10]. The present model adopts these principles but is not presented as a validated PET/CT-specific calculator. Internal arithmetic validation consisted of independent recalculation of debt service, annual cost, and scan-level cost across scenarios and reconciliation of the base operating cost structure. External face validation consisted of checking the required technical and workforce elements against IAEA guidance and comparing the epidemiologic denominator with GLOBOCAN 2022 for Cameroon [5,9,18,19].

### 2.2. Cameroon Case Study and Transferability

Cameroon was used as an illustrative national case because it combines a substantial cancer burden with limited advanced molecular-imaging infrastructure and challenging inter-city logistics. GLOBOCAN 2022 estimated 19,564 new cancer cases in Cameroon, including 4207 breast, 2525 cervical, 2050 prostate, 1480 non-Hodgkin lymphoma, 1148 Kaposi sarcoma, and approximately 916 colorectal cancers [18]. Results are not assumed to be directly transferable to all SSA countries. Transferability depends on income level, geography, pathology capacity, existing nuclear–medicine infrastructure, workforce, regulation, financing arrangements, treatment capacity, and the feasibility of regional referral. It should be noted that GLOBOCAN estimates for Cameroon vary substantially, from 20,745 incident cancer cases in 2020 and 19,564 in 2022 to 16,087 in 2024 despite continued population growth indicating inconsistencies in the GLOBOCAN data itself. However, for our analysis, we have selected 2022 data.

### 2.3. Entry-Level Technology Configuration

For this study, an “entry-level cyclotron-based PET/CT center” was defined as a single-scanner, in-house radiopharmaceutical production facility initially focused on 18F-FDG, with physical and regulatory provisions for later tracer expansion. The modeled technical configuration included:

- one PET/CT scanner suitable for routine whole-body oncology imaging;
- one 11–13 MeV negative-ion medical cyclotron. IAEA guidance identifies beam energy and beam current as key selection parameters; commercial systems in this energy range commonly have nominal proton currents near 100 µA, whereas routine liquid-target operation may be lower because target heat removal limits usable current [5];
- dual-beam capability where available, with at least two 18O-water targets to provide redundancy and scheduling flexibility;
- two automated nucleophilic 18F synthesis modules, preferably allowing a second synthesis, while the first line is unavailable or undergoing cleaning/maintenance; IAEA guidance notes that the ability to perform a second synthesis is advantageous in clinical programs [5];
- shielded hot cells for synthesis/dispensing and a separate quality-control laboratory with an activity calibrator, chromatographic testing capability, pH, residual-solvent analysis, radionuclidic identity/purity testing, bacterial endotoxin testing, and sterility-release procedures appropriate to local regulation;
- radiation monitoring, controlled areas, radioactive-waste storage, HVAC/pressure control appropriate to the radiopharmacy design, uninterrupted power, backup generation, cooling, information systems, and maintenance support.

The initial design corresponds most closely to an IAEA Type 2 facility (cyclotron production for in-house use), with future conversion toward a distribution model only if geography, regulation, production capacity, and quality systems support off-site supply [5].

### 2.4. Capital Cost and Price Year

The details of the capital cost requirement are shown in Table 1. The USD 9 million capital investment used in this analysis represents a planning base case rather than a vendor quotation. This estimate falls within the range implied by the component costs reported in IAEA Technical Reports Series No. 471, after allowing for differences in facility configuration as TRS 471 cost estimates are historical and actual implementation costs would vary according to country, vendor, taxation, freight, shielding requirements, local construction costs, and other site-specific factors. Capital-cost sensitivity analyses of −25%, +25%, and +50% were performed.

### 2.5. Financing Equations and Cost Architecture

For capital principal P, annual interest rate r, and financing term n years, annual debt service (ADS) was calculated using the standard annuity equation:

*ADS* = *P* × [*r*(1 + *r*)*^n^*/((1 + *r*)*^n^* − 1)] (1)

Operating expenditure was divided into fixed costs F, step-fixed staffing costs S(Q), and variable costs vQ, where Q is the number of completed scans:

*OPEX(Q)* = *F* + *S*(*Q*) + *vQ* (2)

*Total annual economic cost*(*Q*) = *ADS* + *OPEX*(*Q*) (3)

*Economic cost per completed scan* = *Total annual economic cost*(*Q*)/*Q* (4)

The public fiscal contribution, reimbursement tariff, and patient out-of-pocket payment were treated as separate quantities. When government provides the capital as a grant, ADS is a public fiscal contribution rather than a cost that must be recovered through the service tariff. When an operating subsidy is provided, the residual amount to be recovered is:

*Residual recovery requirement* = *Total economic cost* − *public capital contribution* − *operating subsidy* (5)

A reimbursement tariff was not calculated in the example as Cameroon does not have F18-based PET-CT scanner, and therefore no national PET/CT tariff was available.

### 2.6. Activity-Based Operating Cost and Workforce

Annual operating expenditure was estimated using a bottom-up planning approach informed by the operational requirements described in IAEA TRS No. 471 and IAEA guidance for planning and operating clinical PET and cyclotron facilities. Table 2 shows the operating expense and the cost per procedure as used in our analysis. The resulting operating cost now depends on the number of scans, which determines the staffing requirement. The annual operating expenditure represents a base-case planning estimate for our analysis. Individual cost components may vary substantially according to equipment service agreements, staffing structure and local salary scales, radiopharmaceutical production volume, quality-control requirements, electricity reliability, generator use, regulatory requirements, and local procurement conditions. Accordingly, operating expenditure was treated as a model parameter and evaluated through sensitivity and activity-based analyses.

Table 3 shows the staffing matrix we have incorporated into our analysis. The mix should be recalibrated to local scope of practice, working hours, shift patterns, and licensing requirements. IAEA guidance requires continuous availability of an appropriately qualified physician during clinical operation and identifies medical physics, radiopharmacy/radiochemistry, technologist, nursing, engineering, radiation-safety, and administrative functions as core elements of a PET/CT imaging center [5].

### 2.7. Financing and Ownership Scenarios

Capital was modeled over 10 years at 3%, 6%, 10%, and 12% APR (Table 6). Completed scan volumes of 500, 1000, 2000, 3000, and 5000 per year were evaluated. Ownership scenarios included: (i) privately financed capital and operations; (ii) publicly provided capital with operating costs recovered through tariff/reimbursement; and (iii) publicly provided capital plus a 50% operating subsidy. The third scenario was expressed as a fiscal-support scenario rather than as a patient price.

### 2.8. Disease-Specific Demand Model

GLOBOCAN 2022 cancer incidence was grouped by major cancer site [18]. For each group, an explicit planning eligibility fraction was assigned to represent the proportion of incident cases expected to reach a guideline-recognized PET indication in the base scenario (Table 4). These fractions are not observed Cameroon rates, but they are transparent planning inputs reflecting the fact that PET use varies greatly by tumor type, disease stage, recurrence status, and tracer. Sensitivity analysis should vary each fraction when local stage-at-presentation data become available.

Table 4 shows the weighted base-case eligibility number of patients for the FDG-PET/CT program. In total, there are 4190 potentially PET-eligible incident cases before pathology and referral losses. This can be considered as a demand in an ideal case. The PET/CT eligibility percentages are model-based planning assumptions rather than empirically observed utilization rates for Cameroon. They were informed by established clinical indications for FDG PET/CT, including disease staging, suspected recurrence, and treatment-response assessment, and they were applied to GLOBOCAN 2022 incident cases to estimate the potential order of magnitude of national PET/CT demand. A brief description is given in column 5 of Table 4.

*Completed incident-case PET demand* = Σ[*Incidence_i* × *eligibility_i*] × *histologic confirmation* × *referral completion* (6)

The base referral-completion assumption after eligibility was 80%. This model does not include repeat scans, surveillance examinations in prevalent cancer cases, or non-oncology PET indications; therefore, it should not be interpreted as total potential scanner workload.

### 2.9. Availability, Production Yield, and Failed Batches

A separate sensitivity analysis modeled 80%, 90%, and 95% system availability for a 3000-slot annual program. Fixed costs and step-fixed staffing were retained, whereas activity-dependent consumables were linked to completed scans. Radiopharmaceutical production failures were incorporated as a planning reserve rather than hidden within successful-scan throughput. Programs should replace this reserve with their observed batch-failure and synthesis-yield data once operational. The model therefore explicitly recognizes that cyclotron downtime, scanner downtime, failed synthesis, QC rejection, and supply interruptions can increase cost per successful scan.

### 2.10. Regulatory and Reimbursement Context in Cameroon

Radiological practices in Cameroon require authorization and regulatory oversight. The former National Radiation Protection Agency (ANRP) was reorganized and renamed the Autorité de Sûreté Radiologique et de Sécurité Nucléaire (ASRAN) by Decree No. 2024/599 within the national framework established by Law No. 2019/012. The regulator’s functions include authorization of practices using ionizing-radiation sources, inspection, quality-control oversight, radiation protection, and monitoring [20]. A cyclotron/PET program would therefore require staged authorization of the facility, occupational/public radiation protection, radioactive-material handling, waste management, and radiopharmaceutical-production activities, in addition to health-sector/pharmaceutical requirements for product release. The precise division of release responsibility should be confirmed with ASRAN and the competent health/pharmaceutical authorities during implementation.

Cameroon launched Phase I of Universal Health Coverage in 2023 and has expanded enrollment, but the publicly described benefit package remains focused on prioritized services and conditions. PET/CT is not identified as a nationally reimbursed service in the public documents reviewed. Household out-of-pocket spending remains high; World Bank/WHO data report out-of-pocket expenditure equivalent to 67.65% of current health expenditure in 2023 [13,14,19]. For this reason, the model does not assume a national PET/CT reimbursement tariff. Instead, it reports on the economic cost and the residual amount that would need to be financed by government, insurance, employers, donors, or patients.

### 2.11. Generator-Based and Network Alternatives

Alternative PET radiopharmaceutical supply strategies were also considered. In particular, a ^68^Ge/^68^Ga generator can provide access to selected ^68^Ga-labeled tracers without requiring an on-site cyclotron and may therefore reduce the initial infrastructure burden associated with introducing specific PET applications. However, a direct quantitative cost comparison with an ^18^F-FDG cyclotron-based program was not included because the tracer portfolios and clinical indications are not equivalent. Generator economics depend on generator acquisition and replacement costs, radioactive decay over the generator life cycle, elution frequency, labeling yield, kits and precursors, quality-control requirements, staffing, the eligible patient population, and the number of clinically usable doses produced per elution. Accordingly, generator-based ^68^Ga imaging was treated as a tracer-specific strategic option rather than as a direct substitute for a comprehensive cyclotron-supported ^18^F-FDG PET/CT program. Table 5 shows the strategic alternatives to a single cyclotron/PET model.

## 3. Results

### 3.1. Financing Cost

Table 6 shows the annual debt service and the amount paid at the end of the 10-year term. At the 6% base-case rate, annual debt service was USD 1.22 million. Lower-cost concessional finance reduced the amount that needed to be recovered from the health system, whereas 10–12% commercial borrowing increased annual debt service materially from USD 1.06 million to USD 1.59 million for an APR of 12%.

Capital-cost sensitivity analysis (Table 7) was performed for USD 9 million base-case investment by varying the assumed initial capital requirement by ±USD 2 million, corresponding to USD 7 million and USD 11 million. Across these scenarios, financing was modeled over 10 years at annual interest rates of 3%, 6%, 10%, and 12%. At the low-capital scenario of USD 7 million, annual debt service ranged from approximately USD 0.82 million at 3% to USD 1.24 million at 12%. At the high-capital scenario of USD 11 million, annual debt service ranged from approximately USD 1.29 million to USD 1.95 million. These results demonstrate that both the initial capital requirement and the cost of financing materially influence the annual fixed-cost burden of PET/CT implementation.

### 3.2. Activity-Based Operating Expenditure and Cost per Scan

The base-case annual operating expenditure and economic cost by completed scan volume (using 6% financing APR) are presented in Table 8. Economies of scale remained substantial, with the modeled cost per completed scan falling from approximately USD 4311 at 500 scans to USD 598 at 5000 scans.

The data presented in the table represent a scenario in which private capital was used to implement the cyclotron-based PET/CT program, in which the investors may need to recover all their investment.

### 3.3. Effect of Public Capital and Operating Subsidy

Public operating subsidies were modeled to assess the extent to which direct government support could reduce the amount that a PET/CT facility would need to recover through service revenue. The underlying annual operating expenditure (OPEX) was maintained for each throughput scenario, while fixed annual government subsidies of USD 500,000 and USD 1 million were evaluated. For each scenario, the subsidy was deducted from the estimated annual OPEX, and the remaining operating expenditure was divided by the number of completed scans to determine the residual cost-recovery requirement per examination. The results are shown in Table 9. These values represent the amount that would need to be recovered from all remaining revenue sources—including patient payments, insurance reimbursement, employer coverage, or other third-party financing—and should therefore not be interpreted as prescribed patient tariffs. Where the modeled subsidy exceeded annual OPEX, the residual operating-cost requirement was reported as zero rather than as a negative cost. This analysis illustrates how the interaction between government support and PET/CT utilization could influence the affordability and financial sustainability of the service.

### 3.4. Disease-Specific Demand and Pathology/Referral Losses

Tayong et al. [21] reported that only 170 of 592 (28.7%) breast cancer patients presenting to the Cameroon Oncology Center had an available pathology report. At the national level, 2796 cancers were recorded in 2021 by participating pathology and haemato-oncology laboratories, compared with 20,745 incident cancers estimated by GLOBOCAN 2020, corresponding to approximately 13.5% of the estimated annual cancer burden. Although this comparison does not represent a formal national histologic-confirmation rate, since not all diagnostic laboratories were included and GLOBOCAN incidence is modeled, it suggests a substantial gap between the projected cancer burden and cancers documented through pathology-based diagnostic services in Cameroon. Thus, a large proportion of cancers estimated to occur nationally may never enter, or may be lost before entering, a pathway leading to pathological confirmation and subsequent specialist referral.

The effects of histologic confirmation and referral completion on estimated PET/CT demand are summarized in Table 10. At 30% histologic confirmation, the disease-specific model generated approximately 1005 completed incident-case PET/CT examinations annually. This scenario is supported by the local clinical experience reported by Tayong et al. [21]. A more restrictive 10% histologic-confirmation scenario, included to represent severe diagnostic-access limitations at the population level, reduced estimated FDG PET/CT demand to approximately 335 examinations annually. The 10% scenario should therefore be interpreted as a sensitivity scenario rather than an estimate of the actual national histologic-confirmation rate.

Increasing histologic confirmation progressively increased potential PET/CT workload; however, the disease-specific model accounts for the fact that not every histologically confirmed malignancy has an indication for FDG PET/CT. At 100% histologic confirmation and 80% referral completion, the model generated approximately 3351 incident-case PET/CT examinations annually. These estimates exclude surveillance, repeat examinations, assessment of recurrence, and non-oncology indications and therefore represent a conservative estimate of potential total PET/CT utilization. Collectively, these findings demonstrate that pathology capacity and successful referral are major upstream determinants of realized PET/CT demand in Cameroon.

### 3.5. Equipment Availability Sensitivity

Modelling the effect of equipment availability is presented in Table 11. For a fixed planned capacity of 3000 slots, reducing system availability from 95% to 80% increased modeled cost per completed scan from approximately USD 911 to USD 1055. This demonstrates the economic importance of preventive maintenance, spare-parts access, backup power, production redundancy, and service contracts in a single-center national model.

## 4. Discussion

The analysis supports the central premise that PET/CT implementation in SSA is a health system challenge rather than a scanner-acquisition exercise. Its principal contribution is not a claim that a particular capital price guarantees sustainability but a transparent framework showing how financing, pathology, clinical appropriateness, workforce, equipment availability, and referral volume interact to determine the amount that must be financed per completed examination.

Patient throughput remains a dominant determinant of average cost because a cyclotron/PET program contains large fixed and step-fixed costs. Consumables, utilities, quality-control activity, and workforce expand with volume. Consequently, economies of scale remain important since the fixed cost are relatively high. The disease-specific demand model also changes interpretation of national cancer incidence. GLOBOCAN 2022 estimated 19,564 new cancers in Cameroon in 2022 [18], but clinical PET eligibility varies markedly by tumor site and stage. Lymphoma contributes a high proportion of eligible cases, whereas routine FDG PET has a much smaller role in prostate cancer. The framework therefore separates cancer incidence, histologic confirmation, disease-specific clinical eligibility, and referral completion. This makes it possible to replace assumptions with local data as pathology registries and stage distributions improve.

The model places equipment reliability inside the economic analysis rather than only in the limitations. A single-cyclotron/single-scanner national center is vulnerable to downtime. Lower availability spreads fixed financing and maintenance costs over fewer successful examinations. Redundancy in targets, synthesis modules, backup power, preventive maintenance, and access to parts therefore has direct financial value even when it increases upfront expenditure. A 68Ge/68Ga generator can provide a lower-capital route to selected PSMA or somatostatin-receptor imaging, but economics depend on generator activity decay, replacement interval, elution schedule, labeling yield, kits, staffing, and the eligible disease population. Conversely, a cyclotron platform that can support 18F-FDG and later 18F-labeled tracers such as 18F-PSMA.

### 4.1. Regional Hub and Network Strategy

Regionalization can improve utilization, but the network design should distinguish patient referral from radiopharmaceutical distribution. Because of the 110 min half life of 18F and variable road/air logistics, cross-border patient referral to a reliable hub may be more feasible than long-distance tracer distribution in some parts of Central Africa. Within dense metropolitan or inter-city corridors such as in Douala or even Yaoundé, a central cyclotron supplying satellite PET/CT scanners may be feasible since transport time, quality systems, and regulation permit. Shared cyclotron ownership and commercial FDG supply should also be considered where they reduce duplication of capital investment.

### 4.2. Workforce, Regulation, and Operational Readiness

Workforce planning must precede commissioning. A sustainable program requires appropriately credentialed imaging physicians, medical physics/radiation-safety support, PET technologists, radiochemistry/radiopharmacy staff, QC personnel, cyclotron engineering, nursing, administration, and biomedical/IT support. Some functions may be contracted, but responsibility for radiopharmaceutical production, QC review, product release, radiation protection, and clinical reporting must be explicit. In Cameroon, ASRAN is the competent radiation-safety regulator for practices using ionizing radiation; implementation would additionally require coordination with the competent health and pharmaceutical authorities for radiopharmaceutical production and release [20].

### 4.3. Phased Implementation Strategy for Successful Fdg-Pet/ct Program

Phase 1: strengthen biopsy/pathology capacity, tumor boards, cancer registration, referral pathways, and financing arrangements; collect site/stage data to calibrate PET eligibility.

Phase 2: develop regulatory capacity, workforce training, facility design, and—in selected settings—generator-based tracer-specific PET or external FDG supply.

Phase 3: commission an in-house cyclotron/18F-FDG program when demand, financing, staffing, quality systems, and maintenance support are sufficient.

Phase 4: expand to 18F-PSMA and other tracers, satellite distribution where feasible, theranostics, research, and regional training.

### 4.4. Policy Implications

PET/CT investment should be evaluated using lifecycle financing rather than equipment purchase price alone.

Procurement decisions should use local 2026 quotations and explicitly model customs, taxes, freight, construction, service agreements, exchange rates, and downtime risk.

National planning should develop pathology and referral networks before assuming high scanner volumes.

Government capital grants, concessional loans, insurance purchasing, employer contracts, and operating subsidies should be analyzed as distinct financing instruments.

Countries should choose among on-site cyclotron, generator-based PET, satellite PET/CT, commercial FDG supply, and shared cyclotron models according to geography and tracer needs rather than adopting a single template for SSA.

### 4.5. Strengths

First, it integrates technical, clinical, and financing decisions within one reproducible health-system framework. Second, it makes the financial equations and cost behavior explicit, allowing local recalibration. Third, it replaces a single aggregate PET-demand fraction with disease-specific incidence and transparent eligibility assumptions. Fourth, it separates economic cost from fiscal subsidy, reimbursement, and patient payment and incorporates equipment availability and alternative network strategies.

### 4.6. Limitations

This remains a deterministic planning model rather than an empirical cost-effectiveness study. Capital inputs are planning estimates, not binding 2026 vendor quotations, and country-specific procurement can vary materially. The activity-based salary envelopes are model assumptions rather than a Cameroon national wage survey. Disease-specific PET-eligibility fractions are transparent planning parameters derived from typical guideline-recognized clinical use, not observed national eligibility rates; local stage-at-presentation data would improve them. The incident-case demand model excludes surveillance and repeat PET/CT while also assuming that tissue confirmation precedes most oncology PET pathways; both features can under- or over-estimate demand for particular cancers. The availability analysis is simplified and does not separately model scanner failure, cyclotron failure, synthesis yield, QC rejection, or spare-parts lead time. The generator comparison is intentionally qualitative because tracer portfolios and production economics are not clinically equivalent. Finally, Cameroon is an illustrative case, and results should not be generalized across SSA without local calibration, but the results should be generally applicable to most countries in Central Africa or even West Africa that do not offer a public insurance health system.

## 5. Conclusions

Cyclotron-based PET/CT programs in Sub-Saharan Africa may achieve financial sustainability under adequate annual patient throughput, appropriate financing, reliable operations, and affordable health system investment. The present model does not demonstrate empirical economic sustainability; it demonstrates conditional financial feasibility under stated assumptions. Sustainable implementation requires coordinated investment in pathology, evidence-based referral, multidisciplinary treatment capacity, workforce, regulation, maintenance, power resilience, financing, and regional networks. Government ownership or capital support can reduce the amount that must be recovered through service tariffs, while stronger pathology and referral systems can increase appropriate utilization. Generator-based and satellite strategies may be valuable complements in selected settings, and cyclotron platforms provide a pathway to broader 18F tracer portfolios including future 18F-PSMA. The framework is intended as a transparent planning tool that governments and cancer centers can recalibrate with local epidemiology, quotations, wage data, regulatory requirements, and payer policy before committing to investments.

## Figures and Tables

**Table 1:** Base-case capital planning estimate for an entry-level cyclotron-based PET/CT center (constant 2026 US dollars).

Component	USD Million	% Total	Model Note
PET/CT scanner	2.00	22.2	Scanner, workstation, installation/acceptance
11–13 MeV cyclotron	2.50	27.8	In-house 18F production; final specification depends on targets/current
Radiochemistry laboratory	0.80	8.9	Clean areas, synthesis support, dispensing
Hot cells	0.60	6.7	Shielded synthesis/dispensing; two-module workflow
QC laboratory	0.40	4.4	Release testing and QA equipment
Building and shielding	1.50	16.7	Cyclotron vault, PET suite, radiopharmacy and controlled areas
Backup power and UPS	0.30	3.3	Generator/UPS/cooling resilience
IT infrastructure	0.10	1.1	RIS/PACS, dose/production records, cybersecurity
Licensing and commissioning	0.20	2.2	Authorization, acceptance, validation, training
Contingency	0.60	6.7	Procurement/construction uncertainty
Total	9.00	100.0	Planning base case; replace with local quotations before investment

**Table 2:** Operating-cost model by cost behavior.

Category	Cost Behavior	Base Model Input (USD)	Notes
Cyclotron maintenance	Fixed	250,000 ≈ 10% initial cost	Annual service planning allowance
PET/CT maintenance	Fixed	200,000 ≈ 10% initial cost	Annual service planning allowance
Staffing	Step-fixed	140,000–320,000	Increases at defined workload thresholds
Radiochemistry/clinical consumables	Variable	75 per completed scan	Reagents, kits, disposables; failed batches assessed separately
Electricity and utilities	Mixed	90,000 + 15/scan	Base load plus activity-related consumption
Generator/backup power	Variable	25 per scan	Planning allocation in addition to base utilities
QA and regulatory compliance	Mixed	80,000 + 10/scan	Calibration, monitoring, release/QA consumables, inspections
IT support	Fixed	20,000	PACS/RIS/production records/support
Miscellaneous operations	Mixed	80,000 + 20/scan	Waste, cleaning, local logistics, general support

**Table 3:** Illustrative activity-based staffing matrix. FTE = full-time equivalent.

Role	500	1000	2000	3000	5000 Scans/y
Nuclear medicine/imaging physician	1	1	1.5	2	3
Medical physicist/radiation-safety support	0.5	0.75	1	1.5	2
PET technologist	2	2	3	4	6
Radiochemist/radiopharmacist	1	1	1.5	2	3
QC scientist/technologist	0.5	0.5	1	1	1.5
Cyclotron engineer	1	1	1	1.5	2
Nurse	1	1	2	2	3
Administration/data	1	1	1.5	2	2.5
Modeled salary/benefit envelope ($)	140,000	160,000	200,000	240,000	320,000

**Table 4:** Disease-specific incident-case demand inputs for Cameroon.

Cancer Group	GLOBOCAN 2022 Incident Cases	Base-Case PET/CT Eligibility Assumption for Demand Modeling	Eligible Number of Patients	Planning Rationale
Breast	4207	20%	841	Selected advanced staging, suspected recurrence/response assessment
Cervix uteri	2525	25%	632	Selected locally advanced staging, recurrence, treatment planning
Prostate	2050	5% for FDG	103	FDG has limited routine role; PSMA-enabled program analyzed as expansion
Non-Hodgkin lymphoma	1480	80%	1184	High relevance for staging and response assessment
Kaposi sarcoma	1148	10%	115	Selected complex/advanced cases
Colorectum	916	25%	229	Selected metastatic/recurrence evaluation
Other cancers	7238	15%	1086	Composite planning assumption for remaining sites

**Table 5:** Strategic alternatives to a single cyclotron/PET model.

Strategy	Potential Advantage	Key Economic/Operational Constraints	Best-Fit Role
68Ge/68Ga generator + PET/CT	Lower initial infrastructure burden; selected tracers	Generator aging/replacement; limited daily activity; tracer-specific eligible population; QC and radiopharmacy still required	PSMA, DOTATATE, and selected emerging applications
Cyclotron + on-site PET/CT	Broad 18F-FDG platform; future 18F tracer expansion	Highest capital and technical complexity; requires reliable throughput	Comprehensive oncology platform
Central cyclotron + satellite PET/CT	Shares production infrastructure across scanners	Requires short transport times, validated distribution, regulatory authorization, and schedule coordination	Dense urban/regional networks
Commercial FDG supply	Avoids local cyclotron capital	Dependent on reliable external producer, transport time, and customs/regulatory conditions	Markets with nearby supplier
Shared institutional cyclotron	Capital sharing and higher utilization	Governance, scheduling, ownership, and distribution agreements	Academic/health-system consortia
18F-PSMA expansion	Uses cyclotron platform and longer 18F half-life than 68Ga	Requires synthesis module/precursor validation and regulatory approval	Prostate-cancer imaging expansion

**Table 6:** Annual debt service calculated using different APR rates for 9 million investments.

APR	Annual Debt Service (USD)	Total Paid over 10 y (USD)	Total Interest (USD)
3%	1,055,075	10,550,746	1,550,746
6%	1,222,812	12,228,116	3,228,116
10%	1,464,709	14,647,086	5,647,086
12%	1,592,857	15,928,575	6,928,575

**Table 7:** Capital Cost sensitivity analysis between USD 7 million and USD 11 million.

Investment Amount	APR	Annual Debt Service (USD)	Total Paid over 10 Year (USD)	Total Interest (USD)
USD 7 million	3%	820,613	8,206,135	1,206,135
	6%	951,076	9,510,757	2,510,757
	10%	1,139,218	11,392,178	4,392,178
	12%	1,238,889	12,388,891	5,388,891
USD 9 million—base case	3%	1,055,075	10,550,746	1,550,746
	6%	1,222,812	12,228,116	3,228,116
	10%	1,464,709	14,647,086	5,647,086
	12%	1,592,857	15,928,575	6,928,575
USD 11 million	3%	1,289,536	12,895,356	1,895,356
	6%	1,494,548	14,945,475	3,945,475
	10%	1,790,199	17,901,993	6,901,993
	12%	1,946,826	19,468,258	8,468,258

**Table 8:** Base-case annual operating expenditure and economic cost by completed scan volume (using 6% financing APR).

Completed Scans/y	Staff Cost (USD)	OPEX (USD)	Total Annual Economic Cost (USD)	Economic Cost/Scan (USD)
500	140,000	932,500	2,155,312	4311
1000	160,000	1,025,000	2,247,812	2248
2000	200,000	1,210,000	2,432,812	1216
3000	240,000	1,395,000	2,617,812	873
5000	320,000	1,765,000	2,987,812	598

**Table 9:** Distinguishing economic cost, public fiscal contribution, and residual recovery requirement.

Scans/Year	OPEX/Scan, No Subsidy	USD 500,000 Subsidy	USD 1 M Subsidy
500	USD 1865	USD 865	USD 0
1000	USD 1025	USD 525	USD 25
2000	USD 605	USD 355	USD 105
3000	USD 465	USD 298	USD 132
5000	USD 353	USD 253	USD 153

**Table 10:** Incident-case PET/CT demand under varying histologic confirmation rates (80% referral completion after PET eligibility).

Histologic Confirmation	Histologically Confirmed Incident Cancers	PET-Eligible After Disease-Specific Fractions	Estimated Completed Incident-Case PET Scans/y
5%	978	209	168
10%	1956	419	335
20%	3913	838	670
30%	5869	1257	1005
50%	9782	2094	1675
100%	19,564	4189	3351

**Table 11:** Availability sensitivity for a program planned for 3000 annual scan slots (6% financing; staffing maintained at the 3000-scan tier).

System Availability	Completed Scans	Total Annual Cost (USD)	Cost/Completed Scan (USD)
80%	2400	2,530,812	1055
90%	2700	2,574,312	953
95%	2850	2,596,062	911

## Data Availability

All inputs required to reproduce the principal model calculations are reported in the manuscript. Public epidemiologic and health-financing data are available from the cited IARC, WHO, and World Bank sources. No individual-level dataset was generated.
